# Developing acceptable contraceptive methods: Mixed-method findings on preferred method characteristics from Burkina Faso and Uganda

**DOI:** 10.12688/gatesopenres.12953.2

**Published:** 2019-09-10

**Authors:** Aurélie Brunie, Rebecca L. Callahan, Amelia Mackenzie, Simon P.S. Kibira, Madeleine Wayack-Pambè

**Affiliations:** 1 Health Services Research, FHI 360, Washington, DC, USA; 2 Contraceptive Technology Innovation, FHI 360, Durham, NC, USA; 3 School of Public Health, Makerere University, Kampala, Uganda; 4 Institut Supérieur des Sciences de la Population, Ouagadougou University, Ouagadougou, Burkina Faso

**Keywords:** Burkina Faso; Uganda; contraceptive methods; user preferences

## Abstract

**Background**: Unmet need remains high in developing regions. New contraceptive technologies may improve uptake and use. This study examines desirable product characteristics.

**Methods**: We added a module to the female questionnaire of the PMA2020 surveys in Burkina Faso and Uganda and conducted 50 focus group discussions (FGDs) with women, 10 FGDs with men, and 37 in-depth interviews (IDIs) with providers across the two countries. FGDs with women and IDIs with providers included a semi-structured ranking exercise on pre-selected product characteristics.

**Results**: Effectiveness, duration, few side effects, cost, and access were the characteristics most commonly reported as important in choosing a method by survey respondents across both countries. Half or more of women surveyed in each country would like a method that lasts at least one year, while 65% in Burkina Faso and 40% in Uganda said they would use a method causing amenorrhea. Qualitative findings show that women want methods with minimal and predictable side effects. Reactions to increased bleeding were negative, especially in Burkina Faso, but perspectives on reduced bleeding were more mixed. Women and providers preferred methods that are discreet and not user-dependent, and associate duration with convenience of use. Some women in Uganda expressed concerns about the invasive nature of long-acting methods, and cost was an important consideration in both countries. In the ranking exercise, discreet use and few side effects often ranked high, while causing amenorrhea and not requiring a pelvic exam often ranked low.

**Conclusion**: Product development should consider user preferences for success in these settings.

## Introduction

Of the 885 million women of reproductive age who want to avoid a pregnancy in developing regions, 214 million have an unmet need for modern contraception ^1^. This figure includes many women who have ever used a modern contraceptive but discontinued its use. Primary reasons for unmet need are concerns about side effects and health risks, infrequent sex, opposition from partners, and misperceptions about pregnancy risk, all of which could potentially be addressed by adapting current methods or developing new technologies that more adequately meet women’s needs ^2^. If these reasons could be overcome, unintended pregnancies could reduce by as much as 59% ^3^.

There is growing interest by donors in accelerating the development of new contraceptive technologies for women. Calliope, the Contraceptive Pipeline Database, currently lists 109 technologies that are in active development ^4^. However, research and development is slow and expensive, and the contraceptive field is littered with failed or abandoned efforts. For example, though this list may not be exhaustive, Calliope identifies an additional 51 products whose development is currently stalled ^4^. Moreover, successful product development hinges not only on bringing products to the market but also on their acceptability among intended users. To this end, the development of methods that women (and men) want to use requires incorporating users’ needs and preferences into the design of new products early in the process. Evidence that can help establish target product profiles is essential to focus research and development resources on those products that are most likely to fit into users’ lives and be successful. Although they differ in the specific approaches used and span different stages of the development process, several examples of efforts aiming to include user perspectives in the development of contraceptives such as SILCS diaphragms, pericoital contraception, vaginal rings and microarray patches or to elucidate preferred contraceptive features are available in the literature ^5– 12^.

The research presented in this article was conducted in Burkina Faso and Uganda to gauge the perspectives of potential contraceptive users, providers, and decision-makers on six long-acting methods at various stages of development and to gain broader insight into acceptable and desired product characteristics. Findings related to the six methods (a new copper intra-uterine device (IUD), a levonorgestrel intra-uterine system, a new single-rod implant, a biodegradable implant, a longer-acting injectable, and a method of non-surgical permanent contraception) are reported elsewhere ^13^. The findings presented here seek to inform product development more broadly. The two sets of findings are reported separately because they were produced using different methodological approaches to acceptability research (i.e. reaction to specific products vs. perspectives on contraceptive method characteristics) and thereby produce information that is relevant at different stages of the product development process. Specific objectives addressed in this paper are to 1) describe desirable product characteristics, and 2) explore why these characteristics matter to potential users.

## Methods

We conducted a cross-sectional, mixed-method study in Burkina Faso and Uganda. In each country, we added a module of 12 questions to the female questionnaire in Round 4 of PMA2020 surveys and conducted focus group discussions (FGDs) with women and with men and in-depth interviews (IDIs) with providers, as described below.

### Study settings

When this study was conducted in 2016–17, modern contraceptive prevalence among women in union in Burkina Faso was 25% and unmet need 29% ^14^. Implants dominated the method mix (48% of the method mix), followed by injectables (33%) ^14^. In Uganda, 32% of married women were using a modern method, while unmet need was 30% ^15^. Injectables (56% of the method mix) and implants (15% implants) were the most popular methods ^15^. The quantitative and qualitative components of the study were conducted separately, as described below.

The Comité d'Ethique pour la Recherche en Santé in Burkina Faso, Makerere University’s School of Public Health Research and Ethics Committee and the Uganda National Council for Science and Technology in Uganda, and FHI 360’s Protection of Human Subjects Committee in the U.S. approved this study.

### Quantitative component

PMA2020 surveys collect data from a nationally representative sample of households and service delivery points to monitor key health and development indicators, as fully explained elsewhere ^16^. A survey of all women of reproductive age living at sampled households is embedded in the household survey ^16^. Data are collected by a network of trained female resident enumerators using mobile phones. The added module was implemented at the end of the regular female questionnaire. In Uganda, questions were asked of all women who were not using a permanent contraceptive method and said they would be interested in using a new contraceptive product that may become available in the future. In Burkina, questions were asked of all current users of any non-permanent method and of non-users who said they thought they would use contraception in the future. Written consent was obtained prior to implementing the female questionnaire, with additional oral consent collected prior to proceeding with the added module due to its research purpose. Data were collected in April-May 2016 in Uganda and between November 2016 and January 2017 in Burkina Faso.

In the module, women were asked about the characteristics that were most important to them in choosing a method, their preferred method duration, and whether they would choose a method that stopped their period during use (see Table 3 for exact question wording). Data were analyzed descriptively in Stata 14, adjusting for the complex sampling design and unit nonresponse to the female questionnaire with sampling variables and weights provided with the dataset, and methods appropriate for subpopulation analysis. We performed design-adjusted Rao-Scott tests (p<0.05) to compare differences in responses between contraceptive user groups (current users, past users, never users).

### Qualitative component

The qualitative component was conducted in five PMA2020 enumeration areas per country, with one urban enumeration area from the region surrounding the capital and four rural enumeration areas each selected from a different region (Boucle du Mouhoun, East, North, and South-West in Burkina Faso and Central, Eastern, Northern, and Western in Uganda). The study included FGDs with women who were aged 15–17 and married or aged 18–49 regardless of marital status, FGDs with adult men (≥18 years), and in-depth interviews (IDI) with a convenience sample of facility-based family planning providers. For women, separate FGDs were conducted with current contraceptive users (separating long-acting and other methods in Uganda but combining them in Burkina Faso) and women who were not currently using a modern method.

In Burkina Faso, women and men were mobilized through local health centers. In Uganda, women were mobilized by Village Health Teams (VHTs), although nurses at health centers and women themselves helped identify potential participants in a few cases. Men were primarily identified through female FGD participants who agreed to have their partner contacted, with some men also mobilized through VHTs and other male participants. Reflecting the service delivery context in each country, providers in Burkina Faso represented the public sector while those in Uganda covered public and private service delivery channels. We aimed to complete two FGDs with each category of women, one FGD with men, and three (Burkina Faso) or five (Uganda) IDIs with providers per region.

**Figure 1.**
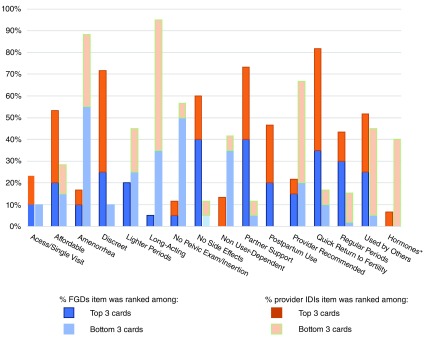
Ranking results for important product characteristics among women and providers in Burkina Faso. *Providers only.

**Figure 2.**
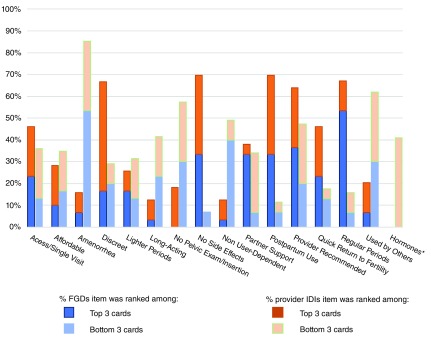
Ranking results for important product characteristics among women and providers in Uganda. *Providers only.

Masters-level research assistants (RAs) conducted all interviews in the local languages (FGDs) or in English or French (providers) in April-May 2016 in Burkina Faso and February 2016 in Uganda. Three topic guides were developed (women, men, providers): each explored participants’ perspectives on and experiences with long-acting reversible contraceptive (LARC) methods, with RAs being instructed to emphasize probing on contraceptive decision-making and bleeding changes in FGDs with women. In FGDs with women and IDIs with providers, these questions were followed by a semi-structured ranking exercise on important product characteristics (see Figure 1 and Figure 2 for full list). Using 15 illustrated cards with women and 16 text-only cards with providers that represented the same concepts (the additional concept for providers was “the method does not contain hormones”), participants were asked to rank the cards in order of importance for women in choosing a method. In FGDs, participants were asked to agree on one final ranking for the group.

FGDs were held in private rooms at pre-arranged locations in the communities, or in a few cases, in a quiet and discrete location outside, while providers were interviewed at their place of work. Written consent was obtained from all participants. On average, FGDs with women lasted 118 minutes, FGDs with men 108 minutes and IDIs with providers 89 minutes. All FGD participants were offered a refreshment and received soap in Burkina Faso and 20,000 shillings (USD 5.40) in Uganda. Providers received a refreshment in Burkina Faso and 12,000 shillings (USD 3.25) in Uganda. All interviews were audio-recorded and translated and transcribed into French or English. Transcripts were uploaded to NVivo 11 for coding and thematic analysis. (All qualitative interviews also included a section exploring perspectives on six products in development; corresponding transcript excerpts were not included in the analysis presented in this paper). Detailed memos described the main dimensions of each main code, and matrices in Excel were used to summarize variations in key themes by country and participant type. Ranking data were analyzed separately for women and providers by computing the frequency at which each card was ranked in the top or bottom three cards.

## Results

### Participant characteristics

A total of 2,743 women in Burkina Faso and 2,403 women in Uganda completed the module added to the PMA2020 surveys; this represented 86% and 63% of all women who completed the main female questionnaire, respectively, and 99.8% and 100% of those eligible for the module. The mean age was close to 27 years old in both countries, and approximately three-quarters of participants were married ( Table 1). The proportions of both current and past contraceptive users were higher in Uganda than in Burkina Faso where 56% of women had never used a modern method.

**Table 1.** Characteristics of respondents to the PMA2020 added module.

Characteristic (%)	Burkina Faso (n=2,743)	Uganda (n=2,403)
**Age, y, mean (SE)**	27.9 (0.3)	27.0 (0.2)
**Parity, mean (SE)**	3.1 (0.1)	3.1 (0.1)
**Residence**		
Urban	23.8	18.2
Rural	76.2	81.8
**Highest education attended**		
None	62.5	6.0
Primary	17.2	64.0
Secondary	18.0	24.6
Higher	2.4	5.4
**Marital status**		
Never married	20.3	17.9
Married/cohabitating	75.9	72.6
Divorced/separated/widowed	3.7	9.5
**Contraceptive use**		
Never user	56.0	37.9
Past user	17.6	25.5
Current modern short-acting user	13.0	27.1
Current modern long-acting user	12.3	6.0
Current traditional user	1.1	3.5

We completed 50 FGDs with women, 10 FGDs with men, and 37 IDIs with providers across the two countries ( Table 2). For FGDs with women, the mean age and parity were similar across participant groups in Burkina Faso (29 years old, 4 children) and Uganda (31 years old, 4 children). Men were 39 years old on average in Burkina Faso and 38 in Uganda; the mean number of children for men was 5 in both countries. In both countries, the mean provider age was 39 and, on average, participants had nine years of experience in their current designation. The sample spanned a range of providers, from physicians to midwives.

**Table 2.** Number of qualitative interviews conducted, by country and participant group.

	Burkina Faso	Uganda
**FGDs with LARC users**		
FGDs	--	10
Participants	--	68
**FGDs with (other) users**		
FGDs	10	10
Participants	79	88
**FGDs with non-users**		
FGDs	10	10
Participants	83	82
**FGDs with men**		
FGDs	5	5
Participants	40	40
**IDIs with providers**		
Public sector	15	13
Private sector	--	9

FGDs, focus group discussions; IDIs, in depth interviews; LARC, long-acting reversible contraceptive.

**Table 3.** Preferences for method characteristics.

User group	Burkina Faso				Uganda			
	Never (n=1,397)	Past (n=509)	Current (n=835)	All (n=2,743)	Never (n=923)	Past (n=593)	Current (n=884)	All (n=2,403)
**Most important characteristics, %** ^a^								
Effectiveness	61.7	62.8	68.8	63.8	51.6	48.2	54.9	51.9
Duration of protection	37.3	33.3	39.0	37.1	31.7	32.8	33.5	32.6
Few other side effects	22.9	39.4	33.2	28.5 ***	26.2	34.8	32.5	30.7 *
Cost	23.5	25.5	24.3	24.1	26.4	23.7	30.1	27.1
Access	20.4	18.5	19.8	19.9	18.6	15.0	23.0	19.3 *
No menstrual bleeding changes	8.0	15.3	11	10.1 ***	14.8	19	21.5	18.3 *
Not painful to receive	6.4	5.0	7.3	6.4	16.7	12.4	16.8	15.6
No effect on sex	2.0	2.8	3.1	2.4	9.9	13.2	15.0	12.6 *
Immediate return to fertility	8.5	9.7	11.8	9.5	8.1	10.0	11.4	9.8
Husband/partner approves	5.0	4.8	5.6	5.2	9.8	9.2	8.9	9.3
Discreet	13.9	10.1	9.7	12.1	9.9	7.6	8.1	8.6
No pelvic exam required	4.4	1.5	4.5	3.9 *	5.7	3.9	4.0	4.6
Compatible with breastfeeding	3.4	1.8	3.6	3.1	4.3	5.4	2.9	4.1
Recommended by friends/ relatives	3.4	5.1	5.2	4.2	1.3	2.0	2.4	1.8
Recommended by provider	1.3	0.9	2.2	1.5	3.8	3.9	2.7	3.4
Available outside clinic	1.2	2.0	3.1	1.9 *	3.0	2.4	3.2	2.9
Other	3.4	3.0	3.0	3.2	6.6	8.3	6.1	6.8
**Preferred method duration, %** ^b^								
Daily	4.4	6.7	7.3	5.6	3.0	1.9	3.2	2.9
Coitally	2.2	2.4	4.2	2.7	3.0	4.4	5.1	4.1
Every month or few months	30.0	28.4	27.8	29.1	32.4	30.9	34.1	32.6
Every year or few years	59.2	55.9	56.2	57.8	50.5	48.5	46.0	48.3
Permanent	2.7	4.7	3.1	3.2	10.4	13.7	11.3	11.6
Other	1.6	2.0	1.5	1.6	0.8	0.7	0.2	0.5
**Willing to use a method** **causing amenorrhea, %** ^c^	64.8	61.7	65.8	64.5	37.9	40.6	41.2	39.8

* p<0.5; ** p<0.1; *** p<0.01

^a^ “In choosing a contraceptive method, what are the things about the method that are important to you?” Multiple responses are possible

^b^ “If you could choose how often to take your contraceptive method, would you choose a method that you would take: every day, every time you have sex, every month or few months, every year or every few years, once (it is permanent), other?”

^c^ “With some contraceptive methods, women do not get their period, but their period and their fertility return when they stop using it. Would you choose a method that stops your period?”

### Results from PMA2020 survey module

Table 3 shows results from questions in the PMA2020 survey module. In both countries, the five characteristics that were most commonly reported as being important in choosing a method were effectiveness, duration of protection against pregnancy, few side effects (besides bleeding changes), cost, and access. Not changing menstrual bleeding was reported as important by 18.3% of women in Uganda and 10.1% of women in Burkina Faso. Other characteristics that were cited by more than 10% of women in Burkina Faso were that the method can be used discreetly, and in Uganda that the method is not painful to receive and does not affect sex. In addition, there were statistically significant differences between user groups. For example, the current and past user groups included higher proportions of women who reported few side effects and no changes in menstrual bleeding as important compared to the never user group in both countries.

Almost three-fifths of women in Burkina Faso and half in Uganda said they would prefer a method that they would take every year or every few years; around 30% of women in each country favored a method that would be taken every month or few months. Among those who preferred methods lasting one or more years, the mean preferred duration was 3.8 years in Burkina Faso and 3.6 years in Uganda (data not shown). The preferred duration for women who wanted methods lasting one or a few months was 3.5 months in Burkina Faso and 3.9 months in Uganda on average (data not shown). In Uganda, 11.6% of women favored permanent methods compared to 3.2% in Burkina Faso.

Overall, 64.5% of women in Burkina Faso and 39.8% of women in Uganda said they would choose a method that stopped their period during use. We found no statistically significant differences between user groups in either country for either preferred method duration or willingness to use a method causing amenorrhea.

### Results from ranking of important product characteristics

Figure 1 and Figure 2 show the proportion of data collection events (summed across FGDs with women and IDIs with providers) within each country in which each characteristic was ranked among the top three and the bottom three cards in the ranking. In both countries, the ability to use the method discreetly and the method not causing side effects like headache or abdominal pain ranked high in over half of data collection events, while causing a woman’s period to stop during use and not requiring a pelvic exam ranked low.

Other characteristics that often ranked high included quick return to fertility, the partner liking the method, the method being less expensive than other methods and friends or relatives recommending the method in Burkina Faso, and the ability to use the method while breastfeeding, having a regular period and the provider recommending the method in Uganda. Other characteristics often ranking low were lasting more than six months and being recommended by a provider in Burkina Faso and being recommended by friends or relatives in Uganda.

### Qualitative results from FGDs with women and with men and IDIs with providers

We collated codes from the analysis of FGD and IDI transcripts into three broader emerging themes to explain data: 1) perspectives on bleeding side effects, 2) perspectives on other product characteristics, 3) characteristics influencing method choice.

***Perspectives on bleeding side effects: All bleeding changes are not perceived equally***. Discussions of side effects primarily revolved around changes in menstrual bleeding. Participants reported negative perceptions of increased bleeding in 17 of 20 FGDs with women and 4 of 5 FGDs with men in Burkina Faso, and in 18 of 30 FGDs with women and 2 of 5 FGDs with men in Uganda. On the other hand, there was little discussion of reduced bleeding by men, and perspectives among women were mixed, though predominantly negative. Overall, many more individual women expressed concerns about increased bleeding as compared to reduced bleeding in the Burkina Faso FGDs. Similarly, many providers in both countries reported observing a range of attitudes related to reduced bleeding among their female clients, but a strong dislike of heavy bleeding. Some women in Uganda and two-thirds of providers in Burkina Faso also mentioned irregular bleeding as an issue for contraceptive users.

A few women in both countries suggested that lighter bleeding was acceptable, but amenorrhea was not. A common concern among women about amenorrhea was that the blood might accumulate in the body. Several women in both countries believed amenorrhea was connected to other side effects, including abdominal pain and swelling in the belly, but also backache in Uganda. In several FGDs in both countries, women suggested the accumulated blood may lead to illness. Some women in Burkina Faso worried about dirt not being flushed out of the body in the absence of menses.

*Every month, there is dirt that must come out of the woman’s body in the form of her period, so if the period comes and this dirt does not come out, over time it is blocked somewhere. If it is blocked, who knows what will happen in the long run? (30-year-old non-user in Burkina Faso, 2342-1)*

In Uganda, a number of participants had concerns about the release of a large quantity of accumulated blood once menses resumed following amenorrhea.

*[When you do not bleed at all] is when you get worried that when it comes, you might bleed to death. This is what we fear. (25-year-old IUD user in Uganda, 1562-5)*

Several women in Uganda and a few providers in both countries noted that amenorrhea could cause women to believe they were pregnant. On the other hand, a few users in Burkina Faso saw the absence of menses as indicative of contraceptive effectiveness, while a few other Burkinabe women agreed that the resumption of menses after use was more important than menstruating during use. **

At times, women linked heavy bleeding to a combination of heavier and prolonged periods, but they often did not explicitly differentiate between the two (or even with irregular bleeding). Women in both countries associated a range of health concerns with heavy bleeding, from weakness or fatigue to dying from massive blood loss. In many cases, women and providers saw heavy bleeding as a condition requiring medical attention. Several women said that more nutritious foods may be needed to counteract blood loss, but often saw improved nutrition as unattainable. A couple of providers in Uganda and a handful in Burkina Faso were specifically concerned about anemia.

*Whenever I over bleed my blood volumes reduce and I also physically lose strength and also you may find that I do not have sufficient foods which are healthy because whenever you bleed you have to eat a good diet so that you can regain your weight and strength. (19-year-old non-user in Uganda, 1242-11)*

Many women and a few providers in both countries described how prolonged bleeding and irregular bleeding interfered with daily life, making them difficult to tolerate. A common concern was the disruption of sex and marital relations, which a few Burkinabe and Ugandan women said could lead men to cheat or even leave their wife.

*If your husband tries to have sex with you, once, twice, three times and that you say it is the time of your period, he will end up saying that you are lying. He will think it is suspicious. Some husbands can even ask you this question: ‘What period flows and never ends?’…you risk packing your bags (your husband can kick you out). (45-year-old Burkinabe non-user who had previously used an implant, 2442-2)*

Other areas of interference somewhat varied based on geography and other participant characteristics. For example, several women in the South-West region of Burkina Faso talked about taboos around cooking while menstruating, while a few Muslim participants said women were not able to pray during their period because they were considered impure. Another concern that was mentioned by several women from different regions in both countries was restricted mobility and fear of getting soiled in public. A 33-year non-user in Burkina Faso who previously used the implant and the injectable said:

*Often when your period comes permanently, when you prepare [it] even, the food does not appeal to you. You cannot go in public, if you sit alone in a corner and people come to find you, you are ashamed to stand because you don’t know if your cloth is stained or not. You cannot go to the market, you cannot go out in public. (33-year-old non-user in Burkina Faso, 2241-10)*

***Perspectives on other product characteristics: Long-acting, user-independent methods that are discreet and non-invasive are more convenient and acceptable***. Overall, methods that are not user-dependent were perceived as more convenient. Whether talking about implants, IUDs, or injectables, many women and providers in both countries noted the convenience (and, relatedly, effectiveness) of these three methods over oral contraceptive pills because they do not require daily intake. Duration and convenience were also interrelated. For example, women in several FGDs emphasized the reduced cognitive and logistical burden of long-acting methods over repeated injections. A Burkinabe woman explained:

*I would prefer what lasts when I am going to want to take. I do not want the tablets or what pricks and three months later you must go again. Maybe the day you must go again to the health center, you could not or you do not have the money. And if you and your husband get near each other, it can be a pregnancy. Me, I do not want that. (33-year-old non-user in Burkina Faso, 2141-1)*

In addition, acceptability is associated with mode of delivery. Participants in half of FGDs with short-acting method users in Uganda and many providers in both countries identified discreet use as an important advantage of injectable contraception.

*Most mothers come for family planning with no consent with their husbands. So, when they are given the injection nobody can know that they have had an injection just for one visit; they get an injection and they go back. (Provider in Uganda, 1223)*

When it comes to devices being inserted, myths around implants or IUDs migrating in the body and concerns that IUDs may negatively affect sex persist among a few women. In Uganda, several FGDs with women not currently using long-acting methods in Uganda also identified concerns with implants and IUDs, including fear of implant insertion and concerns that IUDs could cause cervical cancer.

*With the implant one has to be operated first and when you come to think of it? aaaaaaaah you get scared. Just an injection can make one close the eyes but now the operation is more scaring. (39-year-old injectable user in Uganda, 1252-2)*

***Characteristics influencing method choice: Women seek methods with manageable and predictable side effects, but containing costs remains an important consideration.*** In 18 of 50 FGDs with women, 4 of 10 FGDs with men, and 9 of 37 IDIs with providers across the two countries, participants indicated that cost sometimes overshadows other considerations in choosing a method or acts as a barrier in the ability to start or renew a method, particularly since women sometimes depend on men for access to money. In Uganda, concerns around affordability extended to transportation costs constraining where methods could be obtained.

*For the implant and the IUD, you may need to go to the referral hospital where they will first examine a woman for compatibility then insert it. In such a case, there is need for transport fare and yet at times our partners may not be in a position to give us this money. So, it becomes rather hard, much as we would like to use those methods. (25-year-old injectable user in Uganda, 1252-5)*

Many women and a few men believed that side effects were based on the compatibility of a method with the chemistry of each person’s body (their “blood”). Many FGDs, especially in Uganda, included a discussion of compatibility testing as an element of service provision to determine what method would suit a woman best, and some believed that such “blood tests” already existed.

*When the health workers came on an outreach arrangement, they inserted implants in several women; some found them compatible and yet others failed so they went for removal, so we need health workers to come, provide us with contraceptive counseling, test our blood to realize if our blood [bodies] suits such methods then they do the insertion. (38-year old injectable user in Uganda, 1251-5)*

A number of users described a process of trial-and-error in finding a method with acceptable side effects, and several expressed reluctance to try a different method once they find one that fits their bodies.

*When I came back to use the injectable, I had thought to change with the plastic [implant]. I told myself again that as I am used to the injectable, and I have no problem with it, I am going to take it…so I stayed on that. (30-year old injectable user in Burkina Faso, 2252-4)*

On seeking methods that minimize side effects, a Burkinabe provider noted the advantages of being able to immediately interrupt side effects with implants and IUDs as these methods can be removed:

*This is what makes Jadelle or even the IUD interesting, because if you put [it], in no time if there are problems we can remove. But if it is Depo or Sayana Press, it goes into the blood and there is no removal possible. (2222)*

Many providers found that women tolerate side effects, especially bleeding changes, better when they are forewarned through counseling when they receive the method. In both countries, several women and a few men reported anxiety over the potential added costs of treatment, method removal, or hospitalization particularly in the case of multiple visits or referrals.

*As regards expenditures, it is the implant that is the best…if the one that is inserted in the uterus ends up causing problems to the uterus and you cannot afford to treat her, she can die…if there is a problem, to remove it, it will be necessary to pay. On the other hand, if it is the other, it is not difficult here it can be removed. (42-year old man using condoms in Burkina Faso, 2231-4)*

A couple of women in Uganda highlighted the challenges of affording treatment for women using contraception secretly.

*Our husbands don’t allow us to go for family planning and so some of us just do it in secret and we go. But if you go and then get such a problem of bleeding like a full month and you don’t have the money to buy for you the tablets, those are the effects we fear. (26-year-old non-user in Uganda, 1341-3)*

## Discussion

This research provides insight into important method characteristics for product development from the perspectives of end users in Burkina Faso and Uganda. In both countries, desirable product characteristics include effectiveness, a long duration of action, and few side effects. Avoiding side effects or bleeding changes was comparatively more important for women with prior contraceptive experience than for never users. Qualitative findings from FGDs and IDIs generally support survey results but give additional emphasis to aversion to bleeding changes and discreet use. Both sets of results also highlight cost and access as important considerations. While the ranking exercise did not highlight duration as important (and even suggests it may not be a priority relative to other considerations, especially in Burkina Faso), this apparent discrepancy may be explained by the definition of long-acting that was used in the cards (i.e. lasting more than six months) given that survey results indicate many women would prefer longer durations. Overall, these results are broadly aligned with key concerns linked to unmet need, including side effects, opposition from partners and misperceptions about pregnancy risk. They also highlight the fact in addition to designing product that people will like and want to use, ensuring that these are accessible and affordable will be critical aspects on the continuum from product development to successful introduction.

Half or more of women in each country would like a method that lasts at least a year, with an average preferred duration of more than three years, and close to a third would like a method lasting at least one month with an average preferred duration that would be slightly longer than the current three-month injectable. Approximately 10% of Ugandan women favored permanent methods. Qualitative results show that these preferences are at least partly driven by increased convenience associated with reduced user-dependence and number of clinic visits. Further research is needed to better understand the relative importance of fertility intentions and convenience in preferences for method duration. This is particularly important for new product development as methods allowing for self-use, such as Sayana Press, also have potential to eliminate the challenge of having to visit providers.

Mode of delivery has design implications for contraceptives that directly impact other product characteristics such as duration or side effects. From the perspectives of potential users, however, mode of delivery also has important implications in its own right. The ranking exercise highlighted the ability to use a method without others knowing as important. The opportunities injectables offer for discreet contraceptive use were especially noted in both countries. While longer-acting methods are appreciated, women in Uganda in particular expressed concerns about their invasive nature.

While having few side effects is desirable, participants recognize that side effects are unavoidable with most methods but also are aware that how they manifest varies across individuals. Beyond the importance of counseling on the range of possible side effects associated with a method, our findings draw attention to a desire for more predictability that could be gained through compatibility testing for each potential user. Given that experimentation with different methods and anticipation of costs of treatment and removal both factor into contraceptive decision-making, more effective side effect treatment or reversibility (in the sense of the ability to interrupt use at any time with ensuing and immediate disappearance of side effects) also warrant attention as part of product development and introduction strategies.

Not all side effects were viewed equally, especially when it came to bleeding changes, which can be a cause of contraceptive dissatisfaction, discontinuation, and nonuse ^17^. Our findings also link bleeding changes to method choice. Qualitative results indicate negative attitudes towards heavier bleeding, particularly in Burkina Faso, due to health concerns, anxiety over potential treatment costs, and negative lifestyle implications. On the other hand, perspectives on amenorrhea were mixed. In the PMA2020 survey module, over half of Burkinabe women and two-fourths of Ugandan women said they would use a method causing amenorrhea. However, in the ranking exercise, pausing periods was ranked consistently low in both countries, suggesting that while amenorrhea may be tolerable, it may not be desirable. A secondary analysis further examines women’s perspectives on contraceptive-induced amenorrhea, reporting on the factors associated with acceptability of amenorrhea and on the reasons underlying women’s attitudes ^18^. In addition, the research identified persisting misperceptions, notably linked to health concerns associated with blood accumulating in the body, that should be addressed for existing and future methods.

Finally, our findings reveal that cost and access are pervasive themes. While affordability and availability directly influence economic and geographic access to contraceptive options at uptake, this research is a reminder that they factor more broadly into contraceptive decision-making through financial and practical considerations related to resupply, side effect management, and removal. As advances are being made towards including family planning into insurance schemes to promote universal health coverage schemes, attention should be paid to cover a range of family planning services to alleviate concerns and enhance product acceptability.

### Strengths and limitations

The mixed-method approach and the triangulation of views from providers and clients on important method characteristics with insight gained from actual contraceptive decisions and experiences strengthen confidence in our ability to identify desirable product attributes. Our ability to add a module to the PMA2020 surveys in the two countries allows us to capture the views of nationally representative samples of women. However, the limited number of questions and survey context restrict the breadth and depth of the information that could be collected. Insufficient probing and lack of clarity in qualitative transcripts prevents us from clearly capturing nuances in perspectives on some aspects of bleeding changes such as intensity vs. duration of period or monthly bleeding vs. spotting. The simple ranking exercise offers an additional data point; however, it lacks a measure of difference between items that would allow an exploration of trade-offs. While we were able to gain insight into desirable product characteristics, more research is needed to examine how characteristics can be combined optimally (taking into account both feasibility and acceptability) and how the optimal combination might vary across countries and populations of users.

## Conclusion

Soliciting end-user input early in the product development process is critical to optimize product design and the use of resources and maximize the chances of success. This research provides insight on product characteristics that should be prioritized in product development efforts. While many findings were consistent across Burkina Faso and Uganda, a few were more nuanced and different across the two countries. This reinforces the fact that preferences are part of a larger social and service-delivery context that will ultimately affect demand for new products and deserves careful attention as part of introduction strategies.

## Data availability

### Underlying data

Quantitative survey data are available with PMA2020 household/female datasets for Burkina Faso (Round 4/2016b) and Uganda (Round 4/2016) at the PMA2020 website ( https://www.pma2020.org) or IPUMS PMA website ( https://pma.ipums.org). On both sites, datasets are free to download, but users are required to register and provide a description of the proposed research or analysis. Full qualitative transcripts are not available for ethical reasons because even after removing directly identifiable information such as names and addresses, participant identity may be difficult to fully conceal, and research locations may remain potentially identifiable, presenting a risk of deductive disclosure. However, topic guides, ranking exercise cards and relevant transcript excerpts are available from the authors on reasonable request.
