# Supplementary material for: Developing acceptable contraceptive methods: Mixed-method findings on preferred method characteristics from Burkina Faso and Uganda
Source: Gates Open Res. 2019 Sep 10;3:1205. Originally published 2019 Apr 11. [Version 2] doi: 10.12688/gatesopenres.12953.2 (PMC6963344; doi:10.12688/gatesopenres.12953.2)
Supplement: Peer review history

# Peer review: Reviewer response for version 2

**DOI:** 10.21956/gatesopenres.14201.r27813

**Published:** 2020-01-15

**Authors:** Saumya Ramarao

**Affiliations:** 1 Population Council, New York City, NY, USA

I am satisfied with the authors’ revisions.

I confirm that I have read this submission and believe that I have an appropriate level of expertise to confirm that it is of an acceptable scientific standard.

# Peer review: Reviewer response for version 1

**DOI:** 10.21956/gatesopenres.14058.r27063

**Published:** 2019-07-19

**Authors:** Saumya Ramarao

**Affiliations:** 1 Population Council, New York City, NY, USA

This paper by Brunie *et al.* is a welcome addition to the literature on learning about the perspectives of contraceptive users and potential users about different product attributes so as to inform product design.

 I have listed my comments in order of importance.

1. In terms of methodological innovation, the addition of a module to the PMA2020 to elicit women's perspectives on contraceptive characteristics that were important to them was a good one. The authors complement the PMA2020 survey data with qualitative information. Having said this, I also wanted the paper to compare and contrast this type of methodology and the findings with those used by market research firms. For example, what sorts of biases were avoided or included? Some market research efforts have shown study participants a range of new products and solicited opinions on each. In contrast, in this paper, study participants were not shown products but asked to comment on attributes. It will be important for the authors to elucidate how the combination of PMA2020 and qualitative data shed new or different light. Relatedly, it appears that the ranking of product characteristics was the first step of the qualitative research component. It seemed to have some elements of conjoint analysis; it will be helpful for the authors to flesh this out further - for example, what were the trade-offs that the research participants were willing to make on specific characteristics?
2. Were questions asked of the HIV/STI prevention capability of the contraceptive? This will be important to report given imminent release of the ECHO trial results.
3. It will be helpful if the authors could address the financing of product development and introduction since the purpose of eliciting user input early is to improve the chances of success and better use of resources. Relatedly, since research participants mention the importance of cost considerations in their choice-making, the authors should comment on the policy context of UHC where advances are being made for the inclusion of FP into insurance packages, and other financing mechanisms.

Minor comments follow:

1. Introduction, last paragraph: How do the two papers - the current one and the other – differ? The differences are not clear. Also, please include which six methods are included so that the reader is aware.
2. Results section, first paragraph: 37% of women in Uganda did not complete the product characteristic module. This is a fairly high proportion of drop outs. It will be useful to see if the women who completed the module were different from the women who didn't to examine self-selection.
3. Results from Table 3: "...as being important in choosing a method were effectiveness, duration of protection against a pregnancy, few side-effects, costs and access." The authors should comment on how these align/or not with reasons for unmet need which include side-effects, infrequent sex, opposition from partners, and being postpartum/breastfeeding.  
4. The quantitative data indicate that 64.5% of women in Burkina Faso and 39.8% of Ugandan women would choose a method that stopped their periods. However, the qualitative data do not support this. Were there differences in how the questions were asked/broached in the quantitative and qualitative phases? If possible, it will be good for the authors to include the FGD and IDI discussion guides.
5. Were the questions in the quantitative and qualitative components similar in terms of the questions, sequencing, and how they were raised? It seems that this could explain why some of the data do not triangulate well, especially on amenorrhea.
6. Perspectives on product characteristics - finding that methods not user-dependent were perceived to be convenient. This will be a function of the knowledge that women have of the current methods that are in the program, and not necessarily of new products.
7. It will be useful to include references from other efforts to include user perspectives - notably the SILCS diaphragm, pericoital contraception, and vaginal rings.

I confirm that I have read this submission and believe that I have an appropriate level of expertise to confirm that it is of an acceptable scientific standard, however I have significant reservations, as outlined above.

# Response

**Published:** 2019-09-10

**Authors:** Aurélie Brunie

**Affiliations:** FHI 360, USA

Thank you for taking the time to review and for these comments. Edits were made to the text in response to this feedback and some direct responses are also provided below.

 The data presented in this paper were part of a larger study that also solicited opinions on six specific products (comments 1 & 4). The two parts of the study (perspectives on the six products and insight into desired product characteristics) utilize different approaches to acceptability research and produce information that can be relevant at different stages of the product development process. We made some edits to clarify this in the introduction. We also agree that reflecting on the comparative advantages and disadvantages of various methodological approaches to soliciting input for informing product development is valuable; however, it warrants a much longer discussion than what can be incorporated in this paper. We provide some information under limitations. The authors are currently working on compiling our experiences using different approaches across three studies, which we hope will address this comment in more depth.

 We used a simple ranking exercise (comment 1). In comparison with proportional ranking or discrete choice experiments, this method is limited in its ability to support analysis of trade-offs because it does not include a measure of difference between items. We added a note on this under limitations.

 We tried addressing the value of acceptability research in the broader context of product financing in the introduction and added some text in the conclusion to reinforce this point (comment 3). Some text was also added to the discussion regarding UHC. We also commented on how our results relate to reasons for unmet need (comment 6).

 Thank you for catching the error in wording regarding the proportion of women who responded to the module (comment 5). These were the proportions of all women who responded to the PMA 2020 female questionnaire. When looking at the proportion of those eligible to answer our module, the proportions who agreed to respond were much higher: 99.8% and 100% respectively.

 Qualitative and quantitative data collection solicit information in different ways (comments 7 & 8). We believe that this makes for a combined, stronger understanding of the topics being addressed but may indeed, in some cases, result in conflicting findings. Related to amenorrhea specifically, we conducted a secondary analysis further exploring the two sets of results. We added a reference to this work in the paper.

 No questions were asked that were related to dual protection (comment 2).

# Peer review: Reviewer response for version 1

**DOI:** 10.21956/gatesopenres.14058.r27061

**Published:** 2019-05-07

**Authors:** Cynthia Woodsong

**Affiliations:** 1 Ipas, Chapel Hill, NC, USA

I very much appreciate the work described in this paper, and look forward to citing it in the future, once it is indexed. The issues the authors present are persistent and persistently overlooked by those seeking to develop new contraceptive methods, as well as increase uptake and continuation of existing methods. The study design, particularly choice of countries and study populations, is excellent. I could fill a page of my commendations and agreements with many points made in this paper. The design, data, insights and conclusions are a significant contribution to the field. I hope this paper gets into the hands of a wide range of people. Some of the conclusions are quite unique, and offer explanations to some deep-seated challenges with contraceptive uptake and continuation.

 I have a few comments and suggestions below.

1. With regard to my responses to the peer review form:
  - The information is "clearly and accurately presented," but it does not provide sufficient citations to the current literature. For example, a citation should be provided for PMA2020, key literature or reviews of previous work on contraceptive attributes and preferences (the Polis review focuses on bleeding disruption, yet this manuscript includes a wider range of side effects as well as attributes and preferences), methodology (e.g., card sorts, integrated analysis, statistical analysis), and innovations in the area of what the authors refer to as "compatibility testing" (e.g., genomics, BMI, etc.). If the authors and/or this publication platform expressly intend to minimize citations, the key citation needed is for PMA2020.
  - The source data are only "partly" available. The survey data are available, and the qualitative are not. The authors provide an appropriate explanation for why qualitative transcripts are not available.
  - I am not qualified to comment on the statistical analysis approach, but it seems to need a bit more and/or citations to the methods/approach used.
2. It would be useful to know a bit more about the types of "private sector service delivery channels." Did this include pharmacies? Nurse-midwives? 
3. I'm curious to know why women weren't asked about their views on the inclusion of hormones in their method choices. In my experience, concerns and perceptions about the presence or absence of hormones is something that women in similar populations express.
4. Did the authors look at possible differences in education level and contraceptive use status?

These points aside, I strongly recommend the paper for indexing.

I confirm that I have read this submission and believe that I have an appropriate level of expertise to confirm that it is of an acceptable scientific standard.

# Response

**Published:** 2019-09-10

**Authors:** Aurélie Brunie

**Affiliations:** FHI 360, USA

Thank you for taking the time to review and for these comments. References include a link to the full description of the PMA methodology; we also added other references to the text. We are not able to make the full qualitative transcripts available for ethical reasons related to the terms of informed consent (see statement on data availability). All providers were facility-based, including those in the private sector. The sample did not include pharmacies. The sample spanned a range of positions, from physicians to midwives. Some edits were made to the text to clarify this. Regarding the comment on hormones, the study had another component (around the six methods, as described in the introduction) which included information on the inclusion of hormones into the description of the products. During the data collectors’ training, we found that directly exploring the issue of the inclusion of hormones was challenging, in part because “hormones” did not translate directly into the local languages, which led to conflation with other things such as experiencing side effects. We agree, however, that the notion of including hormones is qualitatively different and warrants further attention. Our analyses report by contraceptive use.
